# Liver abscess complicated with multiple organ invasive infection caused by hematogenous disseminated hypervirulent *Klebsiella pneumoniae*: A case report

**DOI:** 10.1515/med-2023-0694

**Published:** 2023-04-06

**Authors:** Qingyun Chen, Ye Zhang, Lei Zhang, Shaojing Xian, Linhui Huang, Xiuxiu Ding, Hua Wu, Han Xia, Xuying Yang, Xingjun Cai

**Affiliations:** Department of Respiratory and Critical Care Medicine, Hainan General Hospital, Hainan Affiliated Hospital of Hainan Medical University, Haikou 570311, China; Department of Scientific Affairs, Hugobiotech Co., Ltd., Beijing 100176, China; Clinical Laboratory, Hainan General Hospital, Hainan Affiliated Hospital of Hainan Medical University, Haikou 570311, China

**Keywords:** hypervirulent *Klebsiella pneumoniae*, liver abscess, hematogenous dissemination, multiple organ invasive infection, metastatic infection

## Abstract

Hypervirulent *Klebsiella pneumoniae* (hvKp) causes increasing infections in healthy individuals from the community. In severe cases, it can cause multiple organ infection with invasive metastasis of blood sources, seriously threatening the patients’ life. Rapid and accurate diagnosis of the pathogen becomes the key to timely antibiotic treatment to improve the prognosis. This article reports a case of liver abscess complicated with multiple organ invasive infection caused by hematogenous-disseminated hvKp. *K. pneumoniae* was identified by culture and metagenomic next-generation sequencing (mNGS) using blood and liver abscess drainage fluid. The isolates from the two samples were subsequently identified with high homology (99.999%) by whole genome sequencing. In addition, multiple virulence genes were detected in the two isolates and the string test was positive, indicating hvKp with hypermucoviscosity phenotype. Multiple antibiotic treatments were given. The conditions of the patient were stable but the temperature remained high. Surgical drainage treatment was performed, and the patient’s body temperature immediately dropped to normal. He finally recovered after 6 months of follow-up. mNGS using body fluids can facilitate the rapid diagnosis of pathogens. For hvKp infection, choosing a better antibiotic therapy and receiving surgical drainage can significantly improve the prognosis of the patient.

## 1 Introduction

Hypervirulent *Klebsiella pneumoniae* (hvKp) refers to an evolving pathotype that exhibits stronger virulence than classical *K. pneumoniae* (cKp) [1]. hvKp generally causes severe infections and shows the ability to migrate and disseminate to distant sites of the body [2]. The incidence of metastatic infection caused by hvKp ranges from 8 to 24% [3]. Since its discovery in 1986 [4], hvKp has been reported globally, especially in Asian Pacific Rim [5,6,7]. Compared with cKp, which usually causes pneumonia, urinary tract infection, and bacteremia in immunocompromised patients or those frequently exposed to the hospital setting, hvKp can cause life-threatening community-acquired infections in young and healthy individuals. The mortality rate of patients with hvKp bacteremia can reach 35% or even higher [8].

Timely treatment with effective antibiotics is the key to improve the clinical prognosis of patients with hvKp. Therefore, accurate and rapid pathogen diagnosis is important. Culture and string tests were clinically used to identify hvKp but there are some limitations. For example, the sensitivity of culture is low; a string test has been initially used to define hvKp but only 90% of hvKp isolates exhibited a hypermucoviscosity phenotype [9]. New diagnostic approaches with higher sensitivity and accuracy are needed. Metagenomic next-generation sequencing (mNGS) can achieve the same specificity and higher sensitivity as culture methods for pathogen detection and has now been widely used in clinical practice [10,11]. In addition, whole genome sequencing (WGS) can achieve accurate typing and virulence gene detection of hvKp and predict strain resistance. mNGS combined with WGS performs the potential to facilitate the diagnosis and treatment of hvKp.

This article reports a case of liver abscess caused by hematogenous disseminated hvKp complicated with multiple organ invasive infection. Conventional methods combined with mNGS and WGS were applied to identify the pathogen in blood and liver abscess drainage fluid. The patient recovered after a series of antibiotics.

## 2 Case presentation

**Figure 1:**
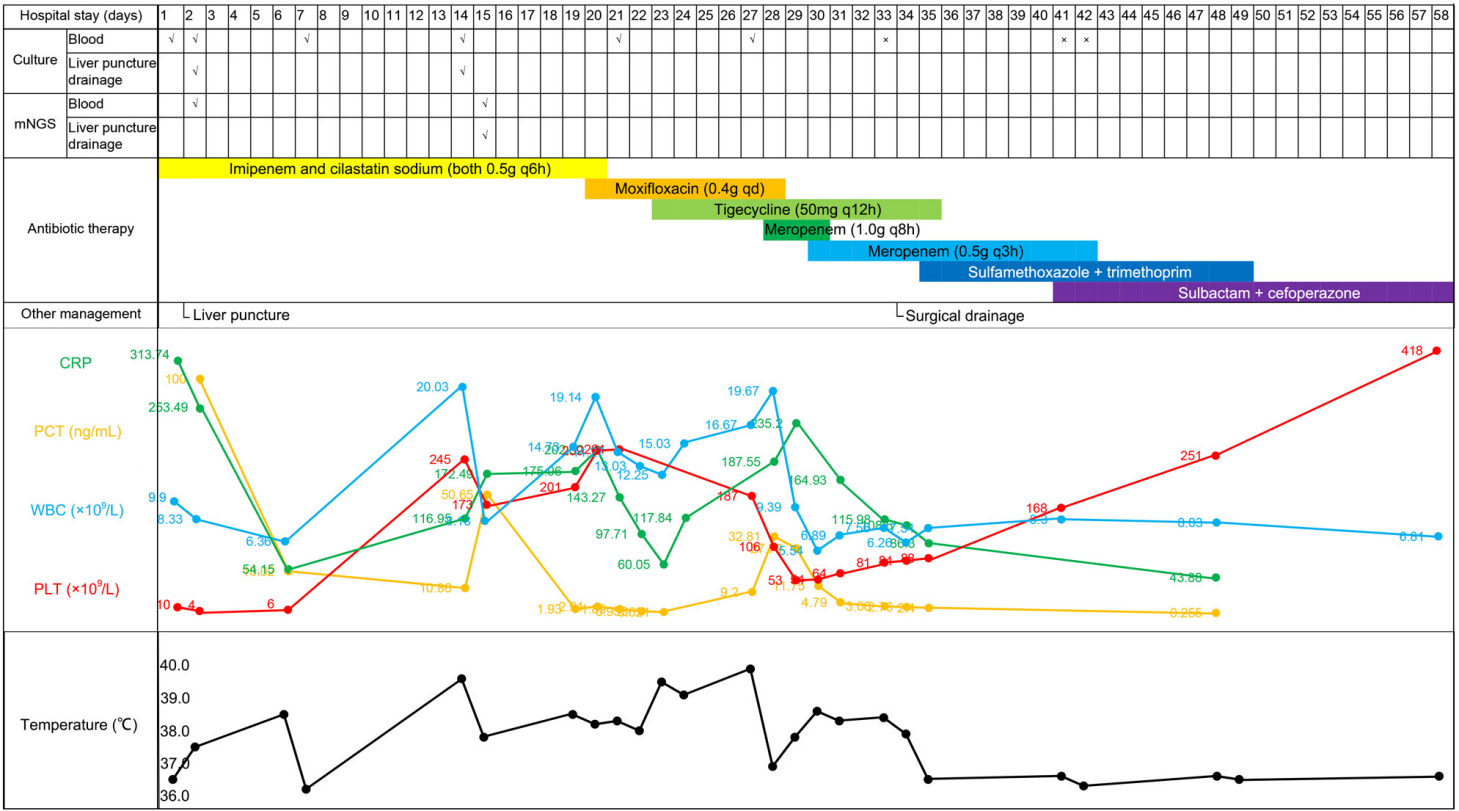
The timeline of the patient. The patient was admitted to the ICU of Hainan General Hospital on October 21, 2021, due to persistent fever and abdominal pain. He was diagnosed with a liver abscess complicated with multiple organ invasive infection caused by hematogenous disseminated hypervirulent *Klebsiella pneumoniae*. After 58 days of hospitalization, the patient was discharged.

A 57 year-old male patient was admitted to the intensive care unit (ICU) of Hainan General Hospital on October 21, 2021, due to persistent fever and abdominal pain (Figure 1). The patient had fever 5 days before admission and complained of epigastric pain 2 days ago. His body temperature fluctuated between 38 and 39°C before admission. The patient had a history of hypertension for 10 years with stable blood pressure control and gout for 10 years with irregular treatment with febuxostat.

**Figure 2:**
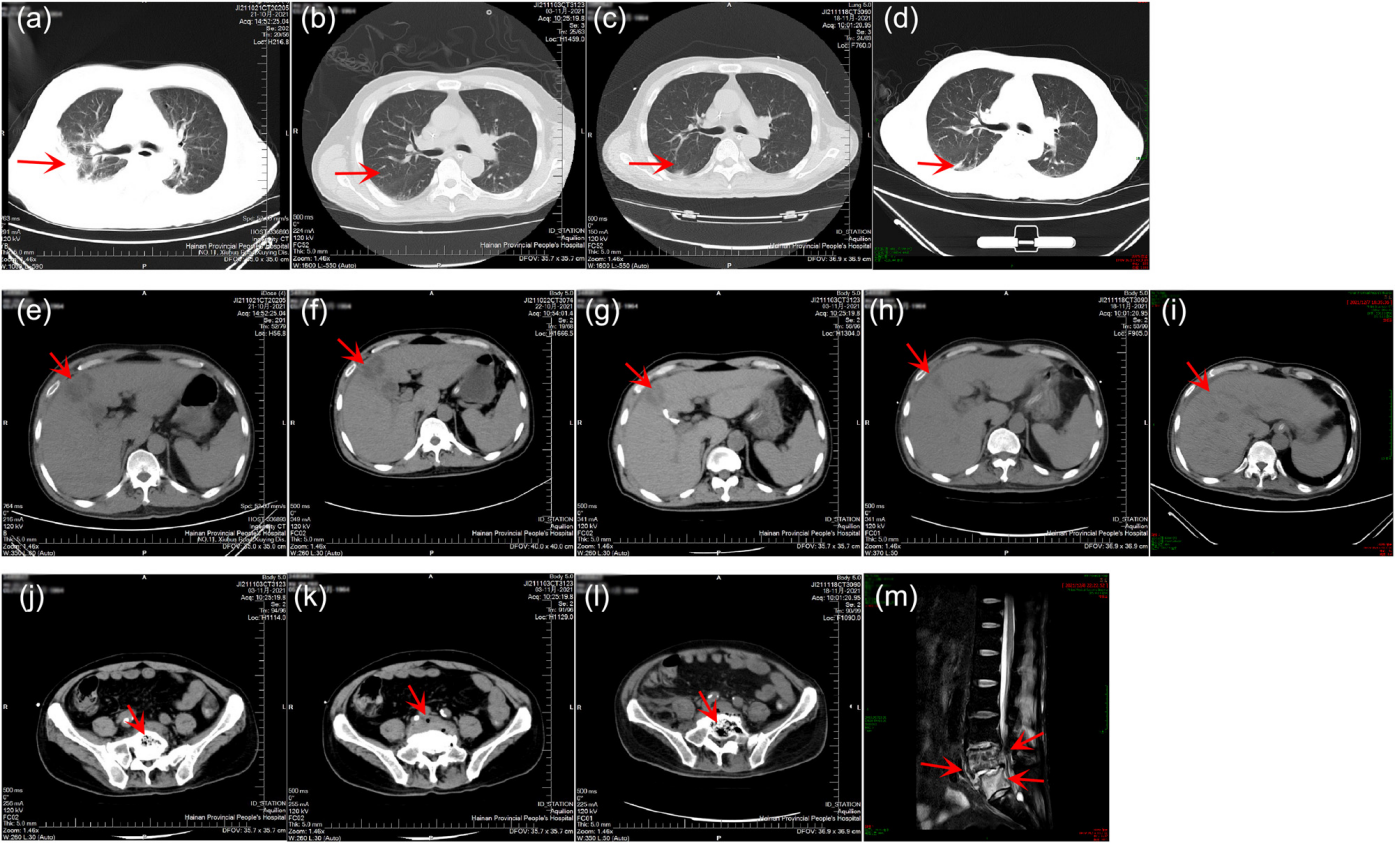
Imaging test results of the patient. (a)–(d) Lung CT results on October 21 (Day 1), November 3 (Day 14), November 18 (Day 29), and December 7 (Day 48), respectively. (e)–(i) Liver CT results on October 21 (Day 1), October 22 (Day 2), November 3 (Day 14), November 18 (Day 29), and December 7 (Day 48), respectively. (j) and (k) Abdominal CT results on November 3 (Day 14). (l) Abdominal CT results on November 18 (Day 29). (m) Lumbar MRI results on December 8 (Day 49).

On the day of admission, his physical examination revealed a body temperature of 36.5°C, a heart rate of 72 beats per min, a respiratory rate of 27 breaths per min, a blood pressure of 110/73 mmHg, disturbance of consciousness and lethargy, coarse breathing in bilateral lungs, moist rales in the right lower lung, and abdominal distention. Blood routine showed white blood cell count (WBC) of 9.9 × 10^9^/L (normal range, 3.5–9.5 × 10^9^/L), decreased platelet count (PLT, 5 × 10^9^/L; normal range, 125–350 × 10^9^/L), and increased blood lactate (LAC, 8.91 mmol/L; normal range, 0.6–2.2 mmol/L). Infection indicator correlation analysis showed increased C-reactive protein (CRP, 312.74 mg/L; normal range, 0–6 mg/L) and procalcitonin (PCT, 100.0 ng/mL; normal range, <0.046 ng/mL). Liver function indicator analysis showed direct bilirubin (D-Bil) of 139.43 μmol/L (normal range, 0–8 μmol/L), indirect bilirubin (I-Bil) of 74.66 μmol/L (normal range, 0–26 μmol/L), and blood creatinine (Scr) of 341 μmol/L (normal range, 57–111 μmol/L). Computed tomography (CT) showed high-density shadows and exudation in the right lung, and quasi-circular low-density lesions with unclear borders in the liver (Figure 2a and e). Suspected vertebral endplate cartilage inflammation was considered but the cause of degeneration could not be ruled out.

**Figure 3:**
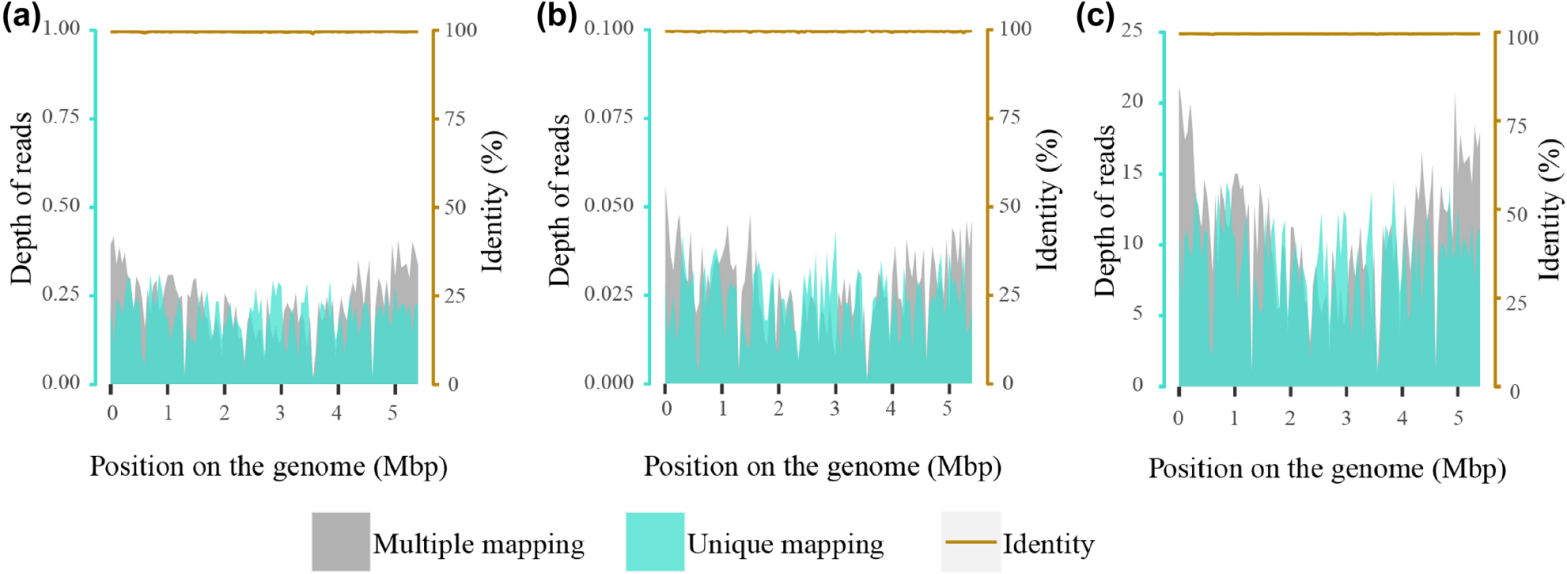
The depth and identity of detected *K. pneumoniae* reads by mNGS using blood on October 22 (Day 2) (a) as well as using blood (b) and liver drainage (c) on November 4 (Day 15). The identity of most reads was >99%.

Enhanced CT on October 22 (Day 2) revealed quasi-circular low-density lesions (2.5 cm × 3.8 cm), indicating liver abscess (Figure 2f). Percutaneous abscess drainage was performed. Culture using blood (Days 1 and 2) and liver abscess drainage fluid (Day 2) was performed, indicating *K. pneumoniae*. A drug susceptibility test was performed and showed no resistance (Table 1). The blood sample (Day 3) was simultaneously collected for mNGS detection, which revealed a total of 19,013 unique reads of *K. pneumoniae* with a genome coverage of 27.51% (Figure 3a). The patient was initially diagnosed with a liver abscess complicated with bloodstream infection and pulmonary infection caused by *K. pneumoniae*. Imipenem combined with cilastatin sodium (both 0.5 g q6h) was given for anti-infective treatment, accompanied by continuous renal replacement therapy and puncture drainage for liver abscess. On November 3 (Day 14), repeated CT showed that the liver abscess was slightly smaller (2.4 cm × 3.0 cm), and suspicious foci of infection in the L5 vertebral body and paravertebral soft tissues were found (Figure 2b, g, and j). Considering the positive *K. pneumoniae* in blood and liver abscess drainage fluid, as well as the subsequent infection in multiple tissues, hvKp was suspected, which has been reported to be more likely to cause liver abscesses with extrahepatic complications [2]. WGS (Hugobiotech, Beijing, China) was therefore performed using the preserved strains from the culture of blood and liver abscess drainage fluid. High homology (99.999%) between the two strains was identified, indicating hematogenous dissemination. Multiple virulence genes were also detected in the two strains, including five important aerobactin-related virulence genes, 17 colibactin-related virulence genes, two LPS-related virulence genes, one *rmpA*-related virulence gene, four Sal-related virulence genes, and 11 Ybt-related virulence genes. The specific plasmid could not be determined due to the incomplete assembly of plasmid sequences. Multilocus sequence typing showed a novel genotype of this isolate. Accordingly, the patient was finally diagnosed with a liver abscess complicated with multiple organ-invasive infection caused by hematogenous disseminated hvKp. Repeated mNGS using blood and liver abscess drainage fluid on November 4 (Day 15) revealed 2,336 and 729,151 unique reads of *K. pneumoniae* (Figure 3b and c), respectively. Though the number of unique reads of *K. pneumoniae* in blood decreased, it remained high in liver abscess drainage fluid. The shortness of breath of the patient was relieved but he still had a fever. The antibiotic treatment continued.

**Table 1:** Susceptibility test results of the liver abscess drainage fluid culture on October 22 (Day 2); drug susceptibility test showed no resistance

Antibiotic	Minimal inhibitory concentration (μg/mL)	Sensitivity
Cefazolin	≤2	Sensitive
Cefuroxime	≤8	Sensitive
Gentamicin	≤1	Sensitive
Cefepime	≤2	Sensitive
Cefoxitin	≤8	Sensitive
Cefatriaxone	≤1	Sensitive
Cefoperazone/sulbactam	≤2/1	Sensitive
Piperacillin/tazobactam	≤4/4	Sensitive
Ticarcillin/clavulanic acid	≤4/2	Sensitive
Ampicillin/sulbactam	≤2/1	Sensitive
Meropenem	≤1	Sensitive
Imipenem	≤1	Sensitive
Amikacin	≤4	Sensitive
Sulfamethoxazole/trimethoprim	≤0.5/9.5	Sensitive
Levofloxacin	≤0.12	Sensitive
Ciprofloxacin	≤0.06	Sensitive
Ceftazidime	≤4	Sensitive
Chloramphenicol	≤8	Sensitive
Minocycline	≤4	Sensitive

On November 9 (Day 20), repeated blood routine showed WBC of 19.14 × 10^9^/L, PLT of 259 × 10^9^/L, CRP of 202.32 mg/L, and PCT of 2.91 ng/mL. The conditions of the patient improved, and the antibiotic was switched to moxifloxacin 0.4 g qd. The patient was transferred to the general ward on November 12 (Day 23). However, his condition deteriorated with a body temperature of 39.5°C, a WBC of 12.25 × 10^9^/L, a PLT of 319 × 10^9^/L, a CRP of 60.05 mg/L, and a PCT of 0.621 ng/mL, and serum creatinine of 141 μmol/L. Tigecycline 50 mg was added for anti-infective treatment. The body temperature was still high, and lethargy occurred. *K. pneumoniae* was still positive by repeated blood culture (Day 27). On November 17 (Day 28), the patient was transferred to the ICU again. Moxifloxacin was switched to meropenem (1.0 g q8h). The condition of the patient improved. The temperature dropped to 37.8°C on November 18 (Day 29). Repeated CT showed that the liver abscess was significantly smaller (2.2 cm × 2.0 cm), but suspicious infectious lesions in the paravertebral soft tissues were unchanged, exudative lesions in the lower lobes of bilateral lungs, around the right psoas muscle, and in the right pelvic wall even developed (Figure 2c, h, and k). The dosage of meropenem was optimized to 0.5 g q3h on November 19 (Day 30).

On November 23 (Day 34), surgical drainage treatment was performed on this patient due to significant swelling of the soft tissue of the right lower limb. The patient’s body temperature dropped to normal on November 24 (Day 35) and the condition gradually improved. Repeated CT before discharge (Day 48) showed infectious lesions of vertebral endplate cartilage and multiple scattered exudations or inflammation of para-vertebral soft tissues, waist muscle, and hip muscle (Figure 2d and i). The patient still suffered exudative lesions in the lower lobes of bilateral lungs but the pleural effusion was basically absorbed. His liver abscess was relieved compared to before but low-density lesions were still observed (Figure 2l and m). After 6 months of follow-up, the patient recovered, with difficulty in walking but no other discomforts.


**Ethics approval and consent to participate:** This study was approved by the ethical review committee of Hainan General Hospital. All procedures performed in studies involving human participants were in accordance with the ethical standards of the institutional and/or national research committee(s) and with the Helsinki Declaration (as revised in 2013). Written informed consent was obtained from the patient for publication of this case report and any accompanying images.

## 3 Discussion

Different from cKp that usually causes invasive hospital-acquired infections in immunocompromised patients [12], hvKp can be acquired in the community, causing infections with higher morbidity and mortality, even in young healthy individuals [13]. Compared to cKp, most patients with hvKp infection suffer liver abscesses, even as the first symptom, with a tendency for metastatic dissemination to other organs, including the eyes, lungs, and the central nervous system. In addition, hvKp can also cause severe infections in the skin, soft tissue, and bone, resulting in necrotizing fasciitis, cervical and psoas abscesses, and osteomyelitis [3,14,15,16,17]. The patient we reported had a history of gout. Liver abscess, accompanied by bloodstream infection and blood-borne disseminated invasive infection involving multiple organs, including pulmonary, bone (lumbar vertebra), and soft tissue (paravertebral, psoas muscle, right lower limb) caused by hvKp was determined in this patient.

A variety of virulence genes were detected in this case, including *rmpA* and *iucA* to *D*. *RmpA* is a typical plasmid-encoded gene that regulates capsular polysaccharide synthesis [18,19]. *IucA* to *D* are associated with the expression of the siderophore system of the strains. The presence of these virulence genes is considered the predictive biomarkers of hvKp in current studies [20]. In addition, other siderophore-associated virulence factors, such as Sal, and yersiniabactin Ybt [21] were also detected in the isolates. The high virulence of the strains may be related to the severe condition of this patient. Previous studies revealed that hvKp can cause community-acquired infection, and diabetes is one of the risk factors for hvKp liver abscess. In this case report, the patient had no history of diabetes. He was admitted to our hospital due to persistent fever and abdominal pain. Blood culture within 24 h after admission revealed *K. pneumoniae*, indicating community-acquired *K. pneumoniae* infection. In addition, *K. pneumoniae* that causes the nosocomial infection is more likely to carry multidrug resistance. However, the susceptibility test results of this case revealed no drug resistance, also suggesting community-acquired *K. pneumoniae* infection of this patient. Though several studies have shown that the hvKp strains infect the liver from the gastrointestinal tract, there was insufficient evidence in this case report. The relationship between this strain and the possible *K. pneumoniae* in the intestine of this patient needs further exploration.

cKp often has multiple drug resistance, and the infection caused by cKp has become a major burden on global public health. Cephalosporins are the main antibiotics for the treatment of abscess caused by *K. pneumoniae* [22], followed by carbapenems, fluoroquinolones, and aminoglycosides [23]. Compared to cKp, hvKp is sensitive to most drugs. However, there have been increasing reports describing hvKp with antibiotic resistance in recent years, including resistance to carbapenems, posing a challenge for clinical treatment [8,24,25]. The drug-sensitive test of this case demonstrated that the isolated hvKp strain was sensitive to these drugs, and carbapenems (such as imipenem and meropenem) showed good effects in this case. However, this patient initially responded only partially to moxifloxacin and tigecycline. Antibiotic treatment for hvKp infection should be based on *in vitro* drug sensitivity tests and clinical drug response. In addition, the patient’s temperature immediately dropped to normal after surgical drainage, indicating its great performance for the treatment of hvKp infection. Thus, choosing appropriate antibiotic therapy of adequate course and receiving focal clearance operation are the key to the treatment of hvKp infection.

In conclusion, mNGS using body fluids can be a potential tool for the clinical diagnosis of pathogens. For patients with suspected hvKp infection, WGS in combination with conventional methods, such as the string test and drug susceptibility test, should be used as quickly as possible to identify the pathogen and guide its antibiotic treatments, improving the prognosis. Surgical drainage combined with an adequate course of antibiotic therapy can significantly improve the conditions of a patient with hvKp infection.
